# starBase v2.0: decoding miRNA-ceRNA, miRNA-ncRNA and protein–RNA interaction networks from large-scale CLIP-Seq data

**DOI:** 10.1093/nar/gkt1248

**Published:** 2013-11-30

**Authors:** Jun-Hao Li, Shun Liu, Hui Zhou, Liang-Hu Qu, Jian-Hua Yang

**Affiliations:** RNA Information Center, Key Laboratory of Gene Engineering of the Ministry of Education, State Key Laboratory for Biocontrol, Sun Yat-sen University, Guangzhou 510275, PR China

## Abstract

Although microRNAs (miRNAs), other non-coding RNAs (ncRNAs) (e.g. lncRNAs, pseudogenes and circRNAs) and competing endogenous RNAs (ceRNAs) have been implicated in cell-fate determination and in various human diseases, surprisingly little is known about the regulatory interaction networks among the multiple classes of RNAs. In this study, we developed starBase v2.0 (http://starbase.sysu.edu.cn/) to systematically identify the RNA–RNA and protein–RNA interaction networks from 108 CLIP-Seq (PAR-CLIP, HITS-CLIP, iCLIP, CLASH) data sets generated by 37 independent studies. By analyzing millions of RNA-binding protein binding sites, we identified ∼9000 miRNA-circRNA, 16 000 miRNA-pseudogene and 285 000 protein–RNA regulatory relationships. Moreover, starBase v2.0 has been updated to provide the most comprehensive CLIP-Seq experimentally supported miRNA-mRNA and miRNA-lncRNA interaction networks to date. We identified ∼10 000 ceRNA pairs from CLIP-supported miRNA target sites. By combining 13 functional genomic annotations, we developed miRFunction and ceRNAFunction web servers to predict the function of miRNAs and other ncRNAs from the miRNA-mediated regulatory networks. Finally, we developed interactive web implementations to provide visualization, analysis and downloading of the aforementioned large-scale data sets. This study will greatly expand our understanding of ncRNA functions and their coordinated regulatory networks.

## INTRODUCTION

Eukaryotic genomes encode thousands of short and long non-coding RNAs (ncRNAs), such as microRNAs (miRNAs), long non-coding RNAs (lncRNAs), pseudogenes and circular RNAs (circRNAs). These RNA molecules are emerging as key regulators of diverse cellular processes, including proliferation, apoptosis, differentiation and the cell cycle (1–7).

Although many studies that address these ncRNAs have focused on defining their protein-coding gene regulatory functions, increasing numbers of researchers are assessing the regulatory interactions between ncRNAs classes, as well as the relationships between RNA-binding proteins (RBP) and ncRNAs. Several well-characterized lncRNAs (e.g. HOTAIR) exert their functions cooperatively with RBPs (e.g. EZH2) in cancers (8,9). Multiple classes of ncRNAs (lncRNAs, circRNAs, pseudogenes) and protein-coding mRNAs function as key competing endogenous RNAs (ceRNAs) and ‘super-sponges’ to regulate the expression of mRNAs in plants and mammalian cells (4,6,7,10–14). However, the understanding of ceRNA mechanisms and its consequences are in their infancy, and further experimental evidences and large-scale bioinformatic efforts for ceRNAs are needed. Despite these intriguing studies of individual miRNA-ncRNA and protein–RNA interactions, generalizing these findings to thousands of RNAs remains a daunting challenge.

Recent advances in high-throughput sequencing of immunoprecipitated RNAs after cross-linking (CLIP-Seq, HITS-CLIP, PAR-CLIP, CLASH, iCLIP) provide powerful ways to identify biologically relevant miRNA-target and RBP–RNA interactions (5,15,16). The application of CLIP-Seq methods has reliably identified Argonaute (Ago) and other RBP binding sites (5,15,16). We and others have used CLIP-seq data generated from HEK293 cells to characterize miRNA-mRNA and miRNA-lncRNA interactions (17–21). With the increasing amount of CLIP-Seq data available, there is a great need to integrate these large-scale data sets to explore the miRNA-pseudogene, miRNA-circRNA and protein–RNA interactions and to further construct ceRNA regulatory networks involving mRNAs and ncRNAs.

**Figure 1.**
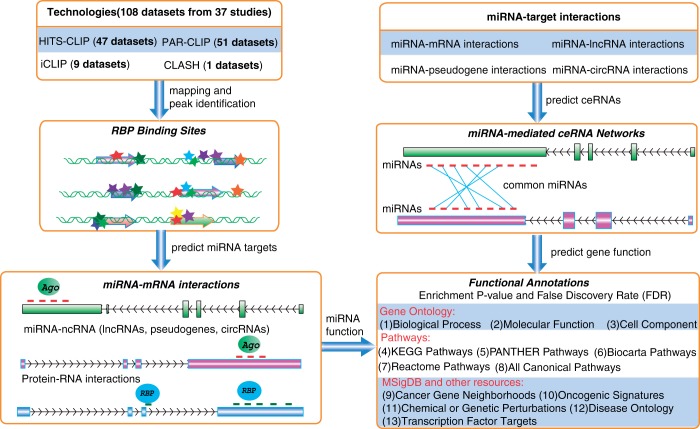
A system-level overview of the starBase v2.0 core framework. A total of 108 data sets of CLIP-seq experiments were compiled to achieve various RBP target sites. Interactions between miRNAs and target genes were predicted and used to construct miRNA-mediated ceRNA networks. Functional predictions of miRNAs and associated genes were achieved by enrichment analysis of 13 functional genomic annotations. All results generated by starBase were deposited in MySQL relational databases and displayed in the visual browser and web pages.

To facilitate the annotation, visualization, analysis and discovery of these interaction networks from large-scale CLIP-Seq data, we have updated starBase (17) to version 2.0 (starBase v2.0) (Figure 1). In starBase v2.0, we performed a large-scale integration of public RBP binding sites generated by high-throughput CLIP-Seq technology and provided the most comprehensive RBP data set for various cell types that are presently available. By analyzing millions of Ago and other RBP binding sites, we constructed the most comprehensive miRNA-lncRNA, miRNA-pseudogene, miRNA-circRNA, miRNA-mRNA and protein–RNA interaction networks.

## MATERIALS AND METHODS

### Integration of Ago and other RBP binding sites from published CLIP data

HITS-CLIP, PAR-CLIP, iCLIP and CLASH data were retrieved from the Gene Expression Omnibus (22), the supplementary data of original references or directly from authors on request (Supplementary Table S1). Although Ago PAR-CLIP raw data were preprocessed with the FASTX-Toolkit v0.0.13 and reanalyzed using PARalyzer v1.1 (23), other CLIP-identified binding sites clusters/peaks were used directly. All binding sites coordinates were converted to hg19, mm9/mm10 and ce6/ce10 assemblies, respectively, by using the UCSC LiftOver Tool (24).

### Ago CLIP-supported miRNA target prediction from public database

Conserved miRNA families were defined as those labeled with ‘highly conserved’ or ‘conserved’ in TargetScan Release 6.2 (25). miRNA IDs from miRBase Release 20 were used (26). Genomic coordinates of these conserved miRNAs target sites predicted by TargetScan (25), miRanda/mirSVR (27), PITA (28), Pictar 2.0 (19) and RNA22 (29) were collected and converted to hg19, mm9/mm10 and ce6/ce10 assemblies using LiftOver, respectively. The resulting coordinates were intersected with the previously described Ago CLIP clusters using BEDTools v2.16.2 (30). The target sites that overlap with any entry of the Ago CLIP clusters were considered as CLIP-supported sites.

### MicroRNA target scanning in annotated transcripts

Human gene annotations were acquired from GENCODE v17 (31). Protein-coding transcripts were defined as those with ‘protein_coding’ gene biotype and ‘protein_coding’ transcript biotype. The lncRNAs transcripts were defined as those with ‘processed_transcript’, ‘lincRNA’, ‘3prime_overlapping_ncrna’, ‘antisense’, ‘non_coding’, ‘sense_intronic’ or ‘sense_overlapping’ gene biotype. Small non-coding RNA (sncRNA) transcripts were defined as those with ‘snRNA’, ‘snoRNA’, ‘rRNA’, ‘Mt_tRNA’, ‘Mt_rRNA’, ‘misc_RNA’ or ‘miRNA’ gene biotype. Pseudogene transcripts were defined as those with ‘polymorphic_pseudogene’, ‘pseudogene’, ‘IG_C_pseudogene’, ‘IG_J_pseudogene’, ‘IG_V_pseudogene’, ‘TR_V_pseudogene’ or ‘TR_J_pseudogene’ gene biotype.

Mouse and *Caenorhabditis elegans* gene annotations were extracted from Ensembl Gene Release 72 and LiftOver to mm9/mm10 and ce6/ce10, respectively. Protein-coding, lncRNAs, sncRNAs and pseudogenes were classified using a similar method. Human, mouse and *C. elegans* circRNA annotations were downloaded from circBase v0.1 (6).

These transcripts were scanned to find conserved miRNAs target sites using miRanda v3.3a with the ‘-strict’ parameter. The target sites that overlap with any entry of the aforementioned AGO CLIP clusters were considered as the CLIP-supported target sites.

### Identification of ceRNA pairs with hypergeometric test

A hypergeometric test (14) is executed for each ceRNA pair separately, which is defined by four parameters: (i) N is the total number of miRNAs used to predict targets; (ii) K is the number of miRNAs that interact with the chosen gene of interest; (iii) n is the number of miRNAs that interact with the candidate ceRNA of the chosen gene; and (iv) c is the common miRNA number between these two genes. The test calculates the *P*-value by using the following formula:

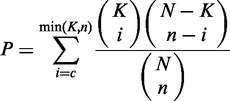

Multiple miRNAs belonging to the same family were combined into one, and the hypergeometric test counted every miRNA family only once, even if it had multiple binding sites at the same 3′-UTR of protein-coding genes or transcript of non-coding genes. All *P*-values were subject to false discovery rate (FDR) correction (32).

### Enrichment analysis for functional terms

GO ontology data (33) for the NCBI RefSeq genes were downloaded from the NCBI ftp site. The Kyoto Encyclopedia of Genes and Genomes (KEGG) pathways (34) were downloaded from the KEGG database. The protein analysis through evolutionary relationships (PANTHER) pathways was downloaded from the PANTHER database (35). The Reactome (36) and other pathways were downloaded from the molecular signatures database (MSigDB) (37). Enrichment analysis for these pathways in the data set was determined using a hypergeometric test with Bonferroni and FDR correction (32).

### Other RBP binding sites in annotated transcripts

The aforementioned RBP CLIP clusters were used to intersect with the coordinates of all annotated transcripts to find their RBP binding sites.

### Other annotation data sets

All refSeq genes were downloaded from the UCSC bioinformatics Web sites (38). Other known ncRNAs were downloaded from the Ensembl database (39) or the UCSC Web sites (38) The human (UCSC hg19), mouse (UCSC mm9/mm10) and *C. elegans* (UCSC ce6/ce10) genome sequences were downloaded from the UCSC bioinformatics Web sites (38).

## DATABASE CONTENT

### The genome-wide binding map of Ago and other RBPs

To depict a comprehensive binding map of Ago and other RBP, we integrated 108 published CLIP-seq data generated from various tissues or cell lines under different treatments in 37 independent studies (detailed in ‘Materials and Methods’, Supplementary Table S1). For the Ago protein, a total of 1 007 618, 26 833 and 4842 unique binding site clusters were compiled in human, mouse and *C. elegans*, respectively (Table 1). These clusters were used in the following analysis to obtain CLIP-supported miRNA target sites of high confidence. Millions of binding site clusters of 42 other RBPs were also achieved (Supplementary Table S1 and Table 1).

**Table 1.** The data sets that are incorporated into starBase v2.0

Species	Experiments	RBPs	Cell lines/ tissues	ABSs	RBSs	miRNA-mRNA	miRNA-ncRNA	ceRNA	protein–RNA
Human	85	36	18	1 007 618	8 206 884	423 975	35 459	11 439	242 017
Mouse	21	11	16	26 833	1 857 199	64 749	234	829	51 542
*C. elegans*	2	2	2	4842	1360	12 883	140	2	411

These statistics show the numbers of sequencing experiments (CLIP-Seq), RNA-binding proteins (RBPs) covered in these experiments, cell lines or tissues used in these experiments, Ago binding sites (ABSs), other RNA-binding protein binding sites (RBSs), miRNA-mRNA interactions, miRNA-ncRNA interactions, ceRNA pairs and protein–RNA interactions that are incorporated into starBase. These data are from three organisms: human (hg19), mouse (mm9) and *C. elegans* (ce6).

### The annotation and identification of miRNA-mRNA and miRNA-ncRNA interactions

To inspect genome-wide interactions between miRNAs and their target genes, we retrieved the conserved miRNA target sites predicted by five algorithms (TargetScan, miRanda, Pictar2, PITA and RNA22) from public databases, which were intersected with the aforementioned Ago CLIP clusters to gain CLIP-supported sites. Using this approach, we characterized ∼500 000 interactions between 818 conserved miRNAs and 20 480 protein-coding genes.

We also investigated the potential regulatory relationships between miRNAs and non-coding RNAs. We performed conserved miRNA target site scanning on the transcripts of lncRNAs, sncRNAs, pseudogenes and circRNAs using miRanda, and filtered the resulting candidates with the previously described Ago CLIP clusters. Although less than CLIP-supported miRNA-mRNA interactions, the thousands of CLIP-supported miRNA-ncRNA interactions suggested that miRNAs might regulate other ncRNAs as well.

### The annotation and identification of miRNA-mediated ceRNA regulatory networks

To construct and characterize the miRNA-mediated ceRNA network, a workflow was developed to identify the ceRNA pairs (Figure 1). First, CLIP-supported miRNA-mRNA, miRNA-lncRNA, miRNA-circRNA and miRNA-pseudogene interactions were combined. Next, hypergeometric test was used to predict ceRNA pairs among mRNAs, lncRNAs, circRNAs and pseudogenes. Finally, all ceRNA pairs with FDR<0.05 were imported into mySQL database and displayed in a web page. In this study, we identified approximately 10 000 ceRNA pairs from CLIP-supported miRNA target sites. Surprisingly, many nodes of ceRNA networks are lncRNAs, circRNAs and pseudogenes. Several experimentally validated ceRNAs were recaptured in our starBase v2.0, e.g. PTEN ceRNA: DCBLD2 (*P* < 0.001), JARID2 (*P* < 0.005), LRCH1 (*P* < 0.00005), TNRC6A (*P* < 0.00005) (12–14).

## WEB INTERFACE

### The web-based exploration of miRNA-mRNA, miRNA-ncRNA and protein–RNA regulatory relationships

Multiple web interfaces are applied to display three types of regulatory relationships. As an example, we explore miRNA-mRNA interactions to introduce the platform application. In the query page of miRNA-mRNA interactions, users can enter a gene symbol and select one miRNA to browse their relationships. The number of supporting experiments can be adjusted to control the stringency of the predictions. All relationships will be displayed in the results page if users do not submit a constraint. Once users click on a non-zero number in the table, more details are shown. For instance, users can click the target location to link to the deepView genome browser (17) and view data across the entire genome (Supplementary Figure S1). More information about the platform application is described in the relevant web interfaces.

### The web-based exploration of ceRNA regulatory networks

In the query page, users enter a gene symbol that is used for ceRNAs prediction. The option of ‘minimum common miRNA number’ denotes the minimum number of miRNAs shared by the input gene and its ceRNA candidates. In the results page, users can click on one of the tabular FDR values for details.

### The web-based functional annotation of genes from miRNA-mediated regulatory networks

For miRNA function predictions, there are five options on the query page, and the option ‘Select one or multiple microRNAs’ is required. In contrast from the options earlier in text, it allows users to select one or more miRNAs in the drop-down list. The results page shows the enrichment analysis for 13 functional prediction categories. The running parameters, selected miRNA target genes and every outcome in the 13 categories of function prediction are available for users to download. For ceRNA functions prediction, the query page also presents users five options and gene symbol is required to enter. The results page is similar to the miRNA function predictions page.

## EXAMPLE APPLICATIONS

In the following section, example applications of starBase v2.0 are illustrated.

### The targetome of hsa-miR-21-5p

Assume that we are interested in the targetome of hsa-miR-21-5p. Given the constraints requiring target sites to have a number of supporting experiments no less than one and to be predicted by at least three of the five programs (Supplementary Figure S2A), our platform returns 173 CLIP-supported hsa-miR-21-5p target sites in 155 protein-coding genes among which ZNF367, RHOB and PELI1 rank top three by read numbers (Supplementary Figure S2B). These results coincide with data in an experimentally validated database named miRTarBase (40), suggesting that these genes are likely targeted by hsa-miR-21-5p.

### The identification of ‘super-sponges’ of miRNAs

Inspired by the observation that CDR1as circRNA (6,7) acts as a miR-7 super-sponge that contains multiple target sites from the same miRNA at the same transcript or 3′-UTR, we tested whether the other class of ncRNAs and protein-coding genes hosted in our database also can act as miRNA super-sponges. We can recapitulate the known CDR1as circRNA as a miR-7 super-sponge using the miRNA-circRNA interactions web page (Supplementary Figure S3A and Supplementary Data). The results page sorted by the number of miRNA target sites showed that CDR1as circRNA contains 52 miR-7 target sites overlapped with CLIP-Seq data (Supplementary Figure S3B). The same strategy was applied to search potential super-sponges among mRNAs, lncRNAs and pseudogenes, resulting in tens of candidates, such as XIST and HOXD-AS1 lncRNA genes and ONECUT2 and CDK6 mRNAs (Supplementary Figure S3C).

### ceRNAs of the oncogenes

Recently, the ceRNA hypothesis has been proposed (3) and efforts have been made to decipher the roles of ceRNA cross talk in regulating cancer-associated genes such as tumor suppressor PTEN (12–14). Requiring a minimum common miRNA number of ten and a FDR threshold of 0.05 on the ‘ceRNA Network’ web page (Supplementary Figure S4A), our platform produced a ceRNA network involving PTEN and 123 genes, among which the published PTEN ceRNAs LRCH1, TNRC6A and SMAD5 (12–14) were recaptured (Supplementary Figure S4B).

We were also able to predict a batch of other cancer-associated genes that were entangled within the highly sophisticated networks of ceRNAs. For example, NFIB, an oncogene upregulated in small cell lung cancer (41) and estrogen receptor-negative breast cancer (42), was found in multiple ceRNA pairs with other well-described cancer-related genes, such as MLL and KDSR (Supplementary Figure S4C).

## CONCLUSIONS

By analyzing a large set of Ago and RBP binding sites derived from all available CLIP-Seq experimental techniques (PAR-CLIP, HITS-CLIP, iCLIP, CLASH), we have shown extensive and complex RNA–RNA and protein–RNA interaction networks.

Compared with the previous version of starBase (v1.0) and other databases, the distinctive features of starBase v2.0 include the following: (i) starBase v2.0 is the first database that provides the miRNA-pseudogene interaction networks; (ii) starBase v2.0 drafts the first interaction maps between miRNAs and circRNAs; (iii) unlike other databases or tools (12,14,43) that predict ceRNA regulatory networks using computationally predicted miRNA targets, starBase v2.0 provides an enhanced resolution to determine ceRNA functional networks based on miRNA-target interactions overlapping with high-throughput CLIP-Seq data; (iv) starBase v2.0 provides the most comprehensive miRNA-lncRNA interactions to date; and (v) starBase v2.0 provides a variety of interfaces and graphic visualizations to facilitate analysis of the massive and heterogeneous CLIP-Seq, RBP binding sites, miRNA targets and ceRNA regulatory networks in normal tissues and cancer cells.

## FUTURE DIRECTIONS

As CLIP-Seq technology is applied to a broader set of species, cell lines, tissues, conditions and RBPs, we will continuously maintain and update the database. starBase will continue to expand the storage space and improve the computer server performance for storing and analyzing these new data, and improve the database to accept new user data uploads. In addition, we intend to integrate the cancer genomics data from the Gene Expression Omnibus (GEO), The Cancer Genome Atlas (TCGA) and International Cancer Genome Consortium (ICGC) into starBase to improve our understanding of miRNA-mediated regulatory networks in developmental, physiological and pathological processes.

## AVAILABILITY

starBase v2.0 is freely available at http://starbase.sysu.edu.cn/. The starBase data files can be downloaded and used in accordance with the GNU Public License and the license of primary data sources.

## FUNDING

Funding for open access charge: Ministry of Science and Technology of China, National Basic Research Program [No. 2011CB811300].

*Conflict of interest statement*. None declared.

## Supplementary Material

Supplementary Data
