# Supplementary material for: starBase v2.0: decoding miRNA-ceRNA, miRNA-ncRNA and protein–RNA interaction networks from large-scale CLIP-Seq data
Source: Nucleic Acids Res. 2013 Nov 30;42(Database issue):D92–7. doi: 10.1093/nar/gkt1248 (PMC3964941; doi:10.1093/nar/gkt1248)
Supplement: Supplementary Data [file gkt1248_supplementary_data.zip › nar-02746-data-e-2013-File002.docx]

**SUPPLEMENTARY MATERIALS**

**starBase v2.0: decoding miRNA-ceRNA, miRNA-ncRNA and protein-RNA interaction networks from large-scale CLIP-Seq data**

**Authors:**

Jun-Hao Li, Shun Liu, Hui Zhou, Liang-Hu Qu* and Jian-Hua Yang*

**Supplementary table and figures**

| Supplementary Table S1 | The 108 CLIP-seq data sets used in this study. |
| --- | --- |
| Supplementary Figure S1 | Illustrative screen shots from the deepView browser. |
| Supplementary Figure S2 | The targetome of hsa-miR-21-5p. |
| Supplementary Figure S3 | Potential miRNA super-sponges. |
| Supplementary Figure S4 | ceRNA prediction for PTEN and NFIB oncogenes. |

**Table S1. The 108 CLIP-seq data sets used in this study**

| **Species** | **Experiment** | **RBP** | **Cell_line / Tissue** | **Treatment** | **Accession** | **Reference** |
| --- | --- | --- | --- | --- | --- | --- |
| Human | PAR-CLIP | AGO2 | hESCs (WA-09) | 4-thiouridine, RNase T1 | Supplementary data | ([1](#_ENREF_1)) |
| Human | HITS-CLIP | AGO2 | HEK293 | completeT1, repA | GSE28865 | ([2](#_ENREF_2)) |
| Human | HITS-CLIP | AGO2 | HEK293 | completeT1, repB | GSE28865 | ([2](#_ENREF_2)) |
| Human | PAR-CLIP | AGO2 | HEK293 | completeT1, repA | GSE28865 | ([2](#_ENREF_2)) |
| Human | PAR-CLIP | AGO2 | HEK293 | completeT1, repB | GSE28865 | ([2](#_ENREF_2)) |
| Human | PAR-CLIP | AGO2 | HEK293 | mildMNase, repA | GSE28865 | ([2](#_ENREF_2)) |
| Human | PAR-CLIP | AGO2 | HEK293 | mildMNase, repB | GSE28865 | ([2](#_ENREF_2)) |
| Human | PAR-CLIP | AGO1 | HEK293 | 4-thiouridine, ML_MM_6 | GSE43573 | ([3](#_ENREF_3)) |
| Human | PAR-CLIP | AGO1 | HEK293 | 4-thiouridine, ML_MM_7 | GSE43573 | ([3](#_ENREF_3)) |
| Human | PAR-CLIP | AGO1 | HEK293 | 4-thiouridine, ML_MM_8 | GSE43573 | ([3](#_ENREF_3)) |
| Human | PAR-CLIP | AGO2 | HEK293 | 4-thiouridine, 3_ML_LG | GSE43573 | ([3](#_ENREF_3)) |
| Human | PAR-CLIP | AGO2 | EF3D-AGO2 | EBV B95-8-infected, 4-thiouridine, RNase T1 | GSE41437 | ([4](#_ENREF_4)) |
| Human | PAR-CLIP | AGO2 | LCL35 | EBV B95-8-infected, 4-thiouridine, RNase T1 | GSE41437 | ([4](#_ENREF_4)) |
| Human | PAR-CLIP | AGO2 | LCL-BAC | EBV B95-8-infected, 4-thiouridine, RNase T1 | GSE41437 | ([4](#_ENREF_4)) |
| Human | PAR-CLIP | AGO2 | LCL-BACD1 | EBV B95-8-infected, 4-thiouridine, RNase T1 | GSE41437 | ([4](#_ENREF_4)) |
| Human | PAR-CLIP | AGO2 | LCL-BACD3 | EBV B95-8-infected, 4-thiouridine, RNase T1 | GSE41437 | ([4](#_ENREF_4)) |
| Human | PAR-CLIP | AGO1 | HEK293 | 4-thiouridine, RNase T1 | GSE21918 | ([5](#_ENREF_5)) |
| Human | PAR-CLIP | AGO2 | HEK293 | 4-thiouridine, RNase T1 | GSE21918 | ([5](#_ENREF_5)) |
| Human | PAR-CLIP | AGO3 | HEK293 | 4-thiouridine, RNase T1 | GSE21918 | ([5](#_ENREF_5)) |
| Human | PAR-CLIP | AGO4 | HEK293 | 4-thiouridine, RNase T1 | GSE21918 | ([5](#_ENREF_5)) |
| Human | PAR-CLIP | AGO2 | HEK293 | miR-124 transfected, 4-thiouridine, RNase T1 | GSE21918 | ([5](#_ENREF_5)) |
| Human | PAR-CLIP | AGO2 | HEK293 | miR-7 transfected, 4-thiouridine, RNase T1 | GSE21918 | ([5](#_ENREF_5)) |
| Human | HITS-CLIP | AGO2 | Hela | control shRNA | GSE42701 | ([6](#_ENREF_6)) |
| Human | HITS-CLIP | AGO2 | Hela | PTB specific shRNA | GSE42701 | ([6](#_ENREF_6)) |
| Human | HITS-CLIP | AGO2 | 293S | arsenite | GSE44404 | ([7](#_ENREF_7)) |
| Human | HITS-CLIP | AGO2 | 293S | hippuristanol | GSE44404 | ([7](#_ENREF_7)) |
| Human | HITS-CLIP | AGO2 | 293S | emetine | GSE44404 | ([7](#_ENREF_7)) |
| Human | HITS-CLIP | AGO2 | 293S | no arsenite | GSE44404 | ([7](#_ENREF_7)) |
| Human | HITS-CLIP | AGO2 | 293S | no hippuristanol | GSE44404 | ([7](#_ENREF_7)) |
| Human | HITS-CLIP | AGO2 | 293S | no emetine | GSE44404 | ([7](#_ENREF_7)) |
| Human | HITS-CLIP | AGO2 | BCBL-1 | KSHV-infected, clusters in 2 of 3 replicates | GSE41357 | ([8](#_ENREF_8)) |
| Human | HITS-CLIP | AGO2 | BC-3 | KSHV-infected, clusters in 2 of 3 replicates | GSE41357 | ([8](#_ENREF_8)) |
| Human | HITS-CLIP | AGO2 | Jijoye | EBV-infected | Supplementary data | ([9](#_ENREF_9)) |
| Human | PAR-CLIP | AGO2 | BC-1 | KSHV-infected, 4-thiouridine | GSE32109 | ([10](#_ENREF_10)) |
| Human | PAR-CLIP | AGO2 | BC-3 | KSHV-infected, 4-thiouridine | GSE32109 | ([10](#_ENREF_10)) |
| Human | CLASH | AGO1 | Flp-In T-REx 293-PTH-AGO1 | CLASH protocol | Supplementary data | ([11](#_ENREF_11)) |
| Mouse | HITS-CLIP | AGO | Brain | 2A8 or 7G1-1* antibody | starBase | ([12](#_ENREF_12)) |
| Mouse | HITS-CLIP | AGO2 | mESCs | WT1A, KO subtracted | GSE25310 | ([13](#_ENREF_13)) |
| Mouse | HITS-CLIP | AGO2 | mESCs | WT1B, KO subtracted | GSE25310 | ([13](#_ENREF_13)) |
| Mouse | HITS-CLIP | AGO2 | mESCs | WT2, KO subtracted | GSE25310, | ([13](#_ENREF_13)) |
| Mouse | HITS-CLIP | AGO2 | CD4+ T cells (C57BL/6) | WT or miR-155 KO | GSE41285 | ([14](#_ENREF_14)) |
| *C. elegans* | HITS-CLIP | ALG-1 | L4-stage worms | WT and alg-1(–) | Supplementary data | ([15](#_ENREF_15)) |
| Human | HITS-CLIP | HuR | HEK293 | completeT1 | GSE28865 | ([2](#_ENREF_2)) |
| Human | PAR-CLIP | HuR | HEK293 | completeT1 | GSE28865 | ([2](#_ENREF_2)) |
| Human | PAR-CLIP | HuR | HEK293 | mildMNase | GSE28865 | ([2](#_ENREF_2)) |
| Human | HITS-CLIP | PTB | Hela | - | GSE42701 | ([6](#_ENREF_6)) |
| Human | PAR-CLIP | IGF2BP1 | HEK293 | doxycycline induced, 4-thiouridine, RNase T1 | GSE21918 | ([5](#_ENREF_5)) |
| Human | PAR-CLIP | IGF2BP2 | HEK293 | doxycycline induced, 4-thiouridine, RNase T1 | GSE21918 | ([5](#_ENREF_5)) |
| Human | PAR-CLIP | IGF2BP3 | HEK293 | doxycycline induced, 4-thiouridine, RNase T1 | GSE21918 | ([5](#_ENREF_5)) |
| Human | PAR-CLIP | PUM2 | HEK293 | 4-thiouridine, RNase T1 | GSE21918 | ([5](#_ENREF_5)) |
| Human | PAR-CLIP | QKI | HEK293 | 4-thiouridine, RNase T1 | GSE21918 | ([5](#_ENREF_5)) |
| Human | PAR-CLIP | TNRC6 | HEK293 | doxycycline induced, 4-thiouridine, RNase T1 | GSE21918 | ([5](#_ENREF_5)) |
| Human | HITS-CLIP | eIF4AIII | Hela | RNase T1, replicate 1 | GSE40778 | ([16](#_ENREF_16)) |
| Human | HITS-CLIP | eIF4AIII | Hela | RNase T1, replicate 2 | GSE40778 | ([16](#_ENREF_16)) |
| Human | HITS-CLIP | DGCR8 | HEK293T | D8 (endogenous DGCR8), replicate 1 | GSE39086 | ([17](#_ENREF_17)) |
| Human | HITS-CLIP | DGCR8 | HEK293T | D8 (endogenous DGCR8), replicate 2 | GSE39086 | ([17](#_ENREF_17)) |
| Human | HITS-CLIP | DGCR8 | HEK293T | T7 (overexpressed DGCR8), replicate 1 | GSE39086 | ([17](#_ENREF_17)) |
| Human | HITS-CLIP | DGCR8 | HEK293T | T7 (overexpressed DGCR8), replicate 2 | GSE39086 | ([17](#_ENREF_17)) |
| Human | PAR-CLIP | FMRP | HEK293 | wildtype FMRP iso1, 4-thiouridine, RNase T1 | GSE39682 | ([18](#_ENREF_18)) |
| Human | PAR-CLIP | FMRP | HEK293 | wildtype FMRP iso7, 4-thiouridine, RNase T1 | GSE39682 | ([18](#_ENREF_18)) |
| Human | PAR-CLIP | FMRP | HEK293 | I304N mutant FMRP iso1, 4-thiouridine, RNase T1 | GSE39682 | ([18](#_ENREF_18)) |
| Human | PAR-CLIP | FMRP | HEK293 | I304N mutant FMRP iso7, 4-thiouridine, RNase T1 | GSE39682 | ([18](#_ENREF_18)) |
| Human | PAR-CLIP | FXR1 | HEK293 | wildtype FXR1, 4-thiouridine, RNase T1 | GSE39682 | ([18](#_ENREF_18)) |
| Human | PAR-CLIP | FXR2 | HEK293 | wildtype FXR2, 4-thiouridine, RNase T1 | GSE39682 | ([18](#_ENREF_18)) |
| Human | HITS-CLIP | FUS | Brain, temporal cortices | replicate1, RNase T1 | GSE43308 | ([19](#_ENREF_19)) |
| Human | HITS-CLIP | FUS | Brain, temporal cortices | replicate2, RNase T1 | GSE43308 | ([19](#_ENREF_19)) |
| Human | HITS-CLIP | FUS | Brain, temporal cortices | replicate3, RNase T1 | GSE43308 | ([19](#_ENREF_19)) |
| Human | PAR-CLIP | LIN28A | HEK293 | 4-thiouridine, RNase T1 | GSE44615 | ([20](#_ENREF_20)) |
| Human | PAR-CLIP | LIN28B | HEK293 | 4-thiouridine, RNase T1 | GSE44615 | ([20](#_ENREF_20)) |
| Human | HITS-CLIP | LIN28 | H9 hESCs | - | GSE39872 | ([21](#_ENREF_21)) |
| Human | HITS-CLIP | LIN28 | LIN28-V5 293 | - | GSE39872 | ([21](#_ENREF_21)) |
| Human | PAR-CLIP | MOV10 | HEK293 | 4-thiouridine | GSE37524 | ([22](#_ENREF_22)) |
| Human | PAR-CLIP | ALKBH5 | HEK293 | 4-thiouridine | GSE38201 | ([23](#_ENREF_23)) |
| Human | PAR-CLIP | C17ORF85 | HEK293 | 4-thiouridine | GSE38201 | ([23](#_ENREF_23)) |
| Human | PAR-CLIP | C22ORF28 | HEK293 | 4-thiouridine | GSE38201 | ([23](#_ENREF_23)) |
| Human | PAR-CLIP | CAPRIN1 | HEK293 | 4-thiouridine | GSE38201 | ([23](#_ENREF_23)) |
| Human | PAR-CLIP | ZC3H7B | HEK293 | 4-thiouridine | GSE38201 | ([23](#_ENREF_23)) |
| Human | PAR-CLIP | EWSR1 | HEK293 | 4-thiouridine | SRA025082 | ([24](#_ENREF_24)) |
| Human | PAR-CLIP | FUS | HEK293 | 4-thiouridine | SRA025082 | ([24](#_ENREF_24)) |
| Human | PAR-CLIP | FUS mutant (FUS-R521G / FUS-R521H) | HEK293 | 4-thiouridine | SRA025082 | ([24](#_ENREF_24)) |
| Human | PAR-CLIP | TAF15 | HEK293 | 4-thiouridine | SRA025082 | ([24](#_ENREF_24)) |
| Human | HITS-CLIP | SFRS1 | HEK293T | - | doRiNA database | ([25](#_ENREF_25)) |
| Human | iCLIP | hnRNP C | Hela | untreated | E-MTAB-1371 | ([26](#_ENREF_26)) |
| Human | iCLIP | U2AF65 | Hela | control siRNA | E-MTAB-1371 | ([26](#_ENREF_26)) |
| Human | iCLIP | U2AF65 | Hela | hnRNP C-specific siRNA or control siRNA | E-MTAB-1371 | ([26](#_ENREF_26)) |
| Human | iCLIP | TIA1 | Hela | - | doRiNA database | ([27](#_ENREF_27)) |
| Human | iCLIP | TIAL1 | Hela | - | doRiNA database | ([27](#_ENREF_27)) |
| Human | iCLIP | hnRNP C | Hela | - | doRiNA database | ([28](#_ENREF_28)) |
| Human | iCLIP | UPF1 | Hela | replicate1, untreated | GSE47976 | ([29](#_ENREF_29)) |
| Human | iCLIP | UPF1 | Hela | replicate2, untreated | GSE47976 | ([29](#_ENREF_29)) |
| Human | iCLIP | UPF1 | Hela | replicate2, puromycoin | GSE47976 | ([29](#_ENREF_29)) |
| Mouse | HITS-CLIP | NOVA | Brain | - | doRiNA database | ([12](#_ENREF_12)) |
| Mouse | HITS-CLIP | FUS | Hlbx9-GFP+ (ES-derived neuronal cells) | replicate1, RNase T1 | GSE43308 | ([19](#_ENREF_19)) |
| Mouse | HITS-CLIP | FUS | Hlbx9-GFP+ (ES-derived neuronal cells) | replicate2, RNase T1 | GSE43308 | ([19](#_ENREF_19)) |
| Mouse | HITS-CLIP | SRSF1 | MEF (SRSF1 tet-repressible) | - | GSE44591 | ([30](#_ENREF_30)) |
| Mouse | HITS-CLIP | SRSF2 | MEF (SRSF2 tet-repressible) | - | GSE44591 | ([30](#_ENREF_30)) |
| Mouse | HITS-CLIP | MBNL2 | hippocampus | - | GSE38497 | ([31](#_ENREF_31)) |
| Mouse | HITS-CLIP | nElavl | forebrain | - | Supplementary data | ([32](#_ENREF_32)) |
| Mouse | HITS-CLIP | FUS | cerebrum | - | GSE37190 | ([33](#_ENREF_33)) |
| Mouse | HITS-CLIP | PTBP2 | embryonic cortex | - | GSE47564 | ([34](#_ENREF_34)) |
| Mouse | HITS-CLIP | MBNL1 | brain (C56BL/6) | - | GSE39911 | ([35](#_ENREF_35)) |
| Mouse | HITS-CLIP | MBNL1 | brain (129SVJ) | - | GSE39911 | ([35](#_ENREF_35)) |
| Mouse | HITS-CLIP | MBNL1 | heart (C56BL/6) | - | GSE39911 | ([35](#_ENREF_35)) |
| Mouse | HITS-CLIP | MBNL1 | muscle (C56BL/6) | - | GSE39911 | ([35](#_ENREF_35)) |
| Mouse | HITS-CLIP | MBNL1 | C2C12 myoblasts | - | GSE39911 | ([35](#_ENREF_35)) |
| Mouse | PAR-CLIP | Cirbp | MEF | 4-thiouridine | GSE40468 | ([36](#_ENREF_36)) |
| Mouse | PAR-CLIP | Rbm3 | MEF | 4-thiouridine | GSE40468 | ([36](#_ENREF_36)) |
| *C. elegans* | iPAR-CLIP | GLD1 | BS1080 | 4SU or 6SG | GSE33569 | ([37](#_ENREF_37)) |

Figure S1. Illustrative screen shots from the deepView browser.


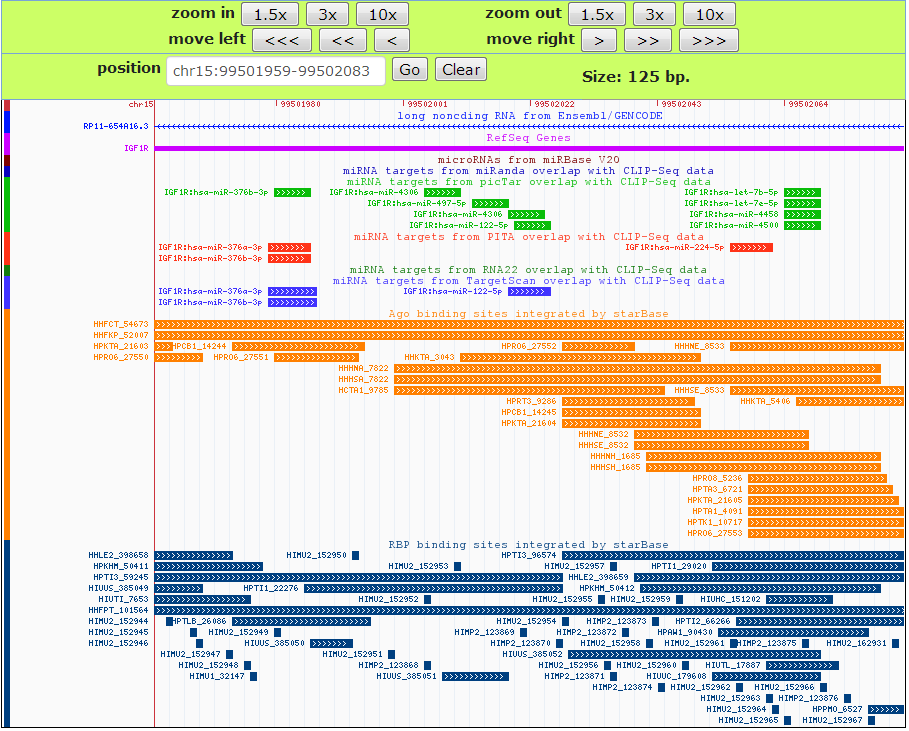


Figure S2A. Studying the targetome of hsa-miR-21-5p in the query page of target site intersections


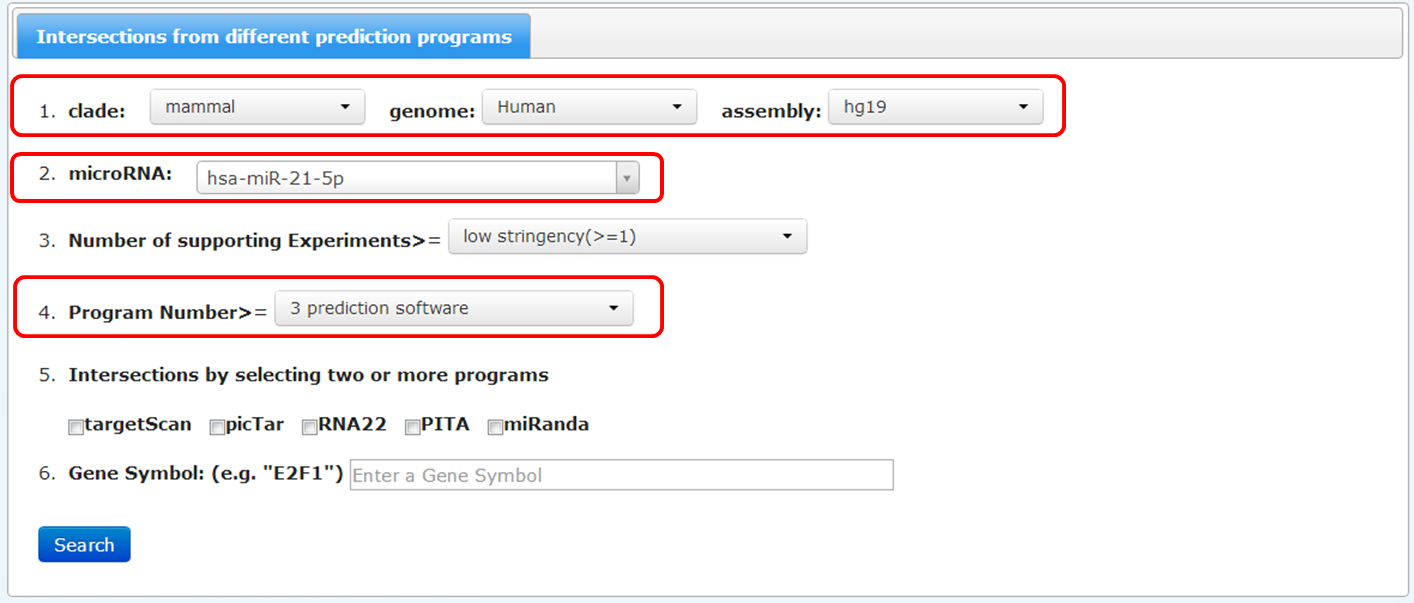


Figure S2B. The results page of the targetome of hsa-miR-21-5p


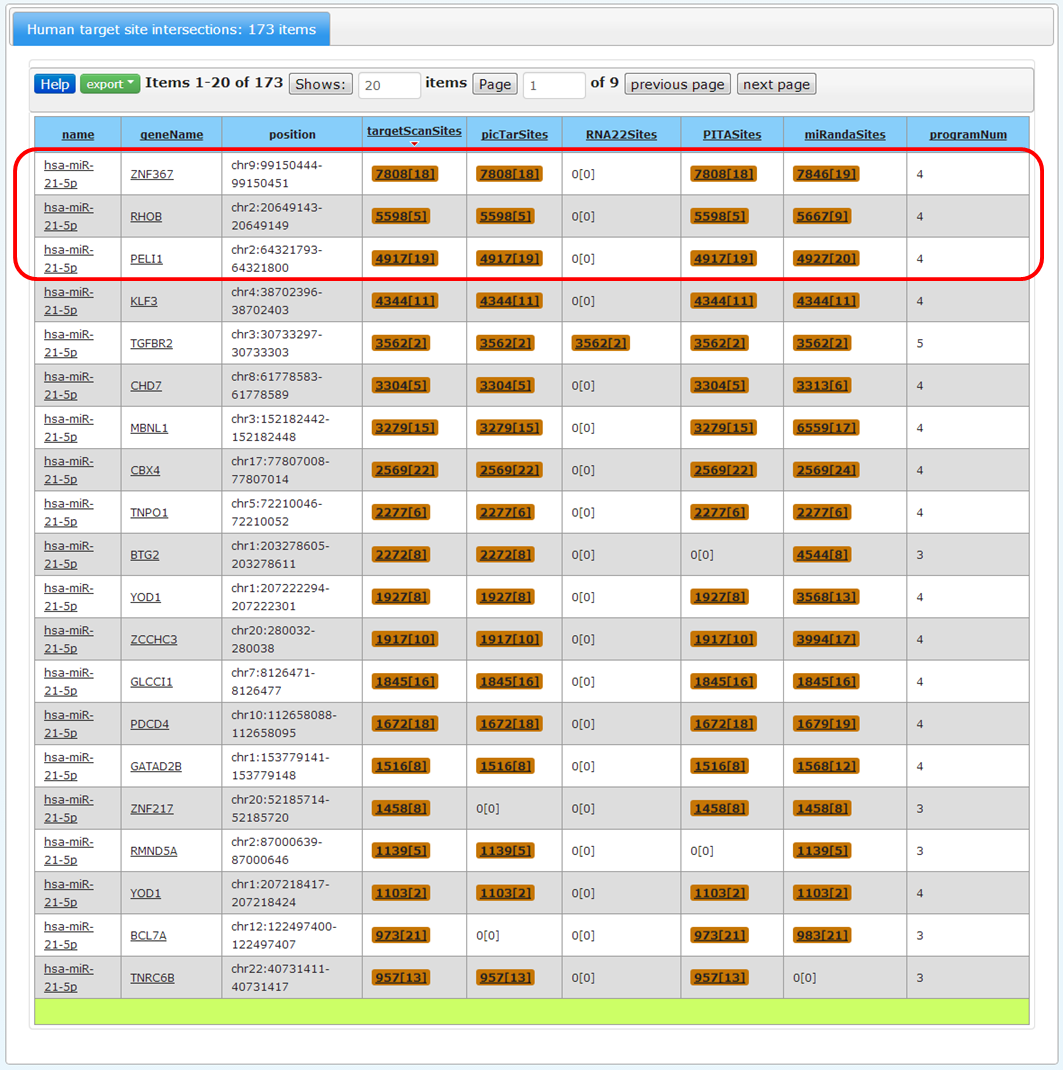


Figure S3A. The identification of super-sponges of miRNAs in the query page of miRNA-circRNA interactions


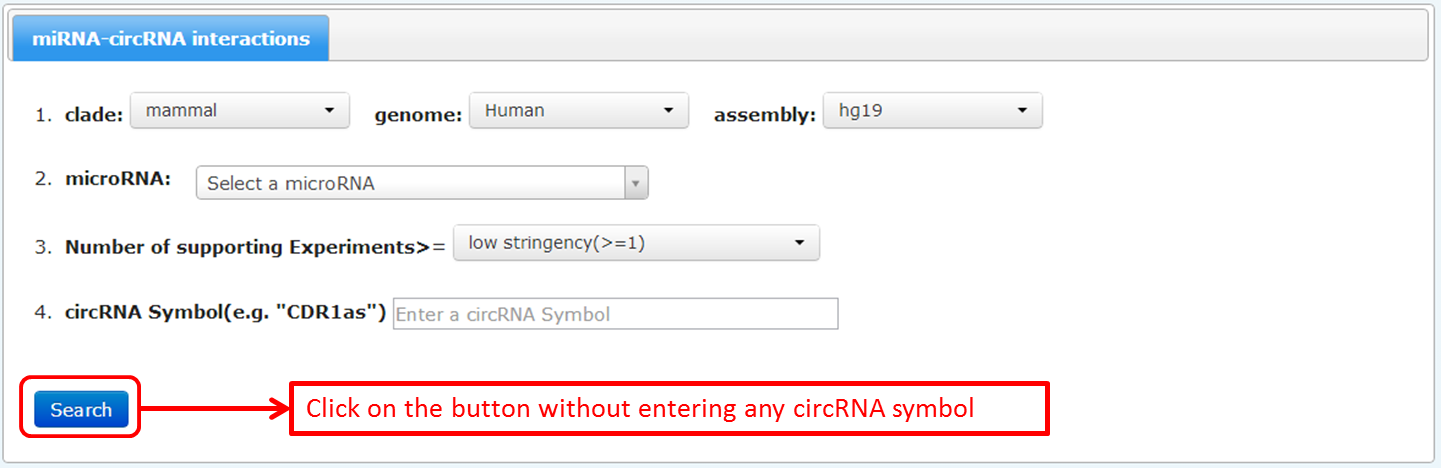


Figure S3B. The circRNA CDR1as as a miR-7 super-sponge in the results page of miRNA-circRNA interactions


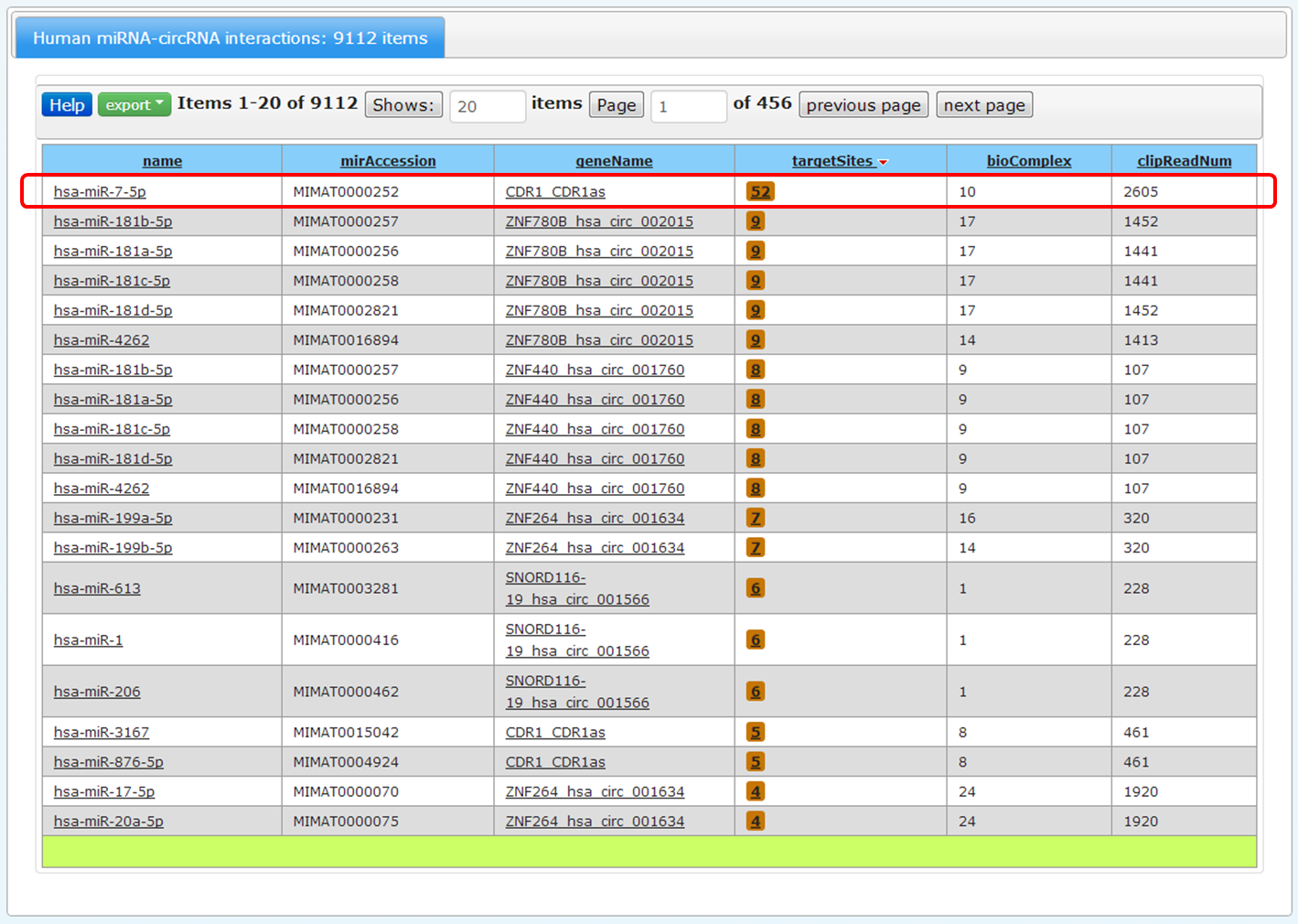


Figure S3C. Some super-sponges candidates among lncRNAs, pseudogenes and mRNAs via starBase v2.0


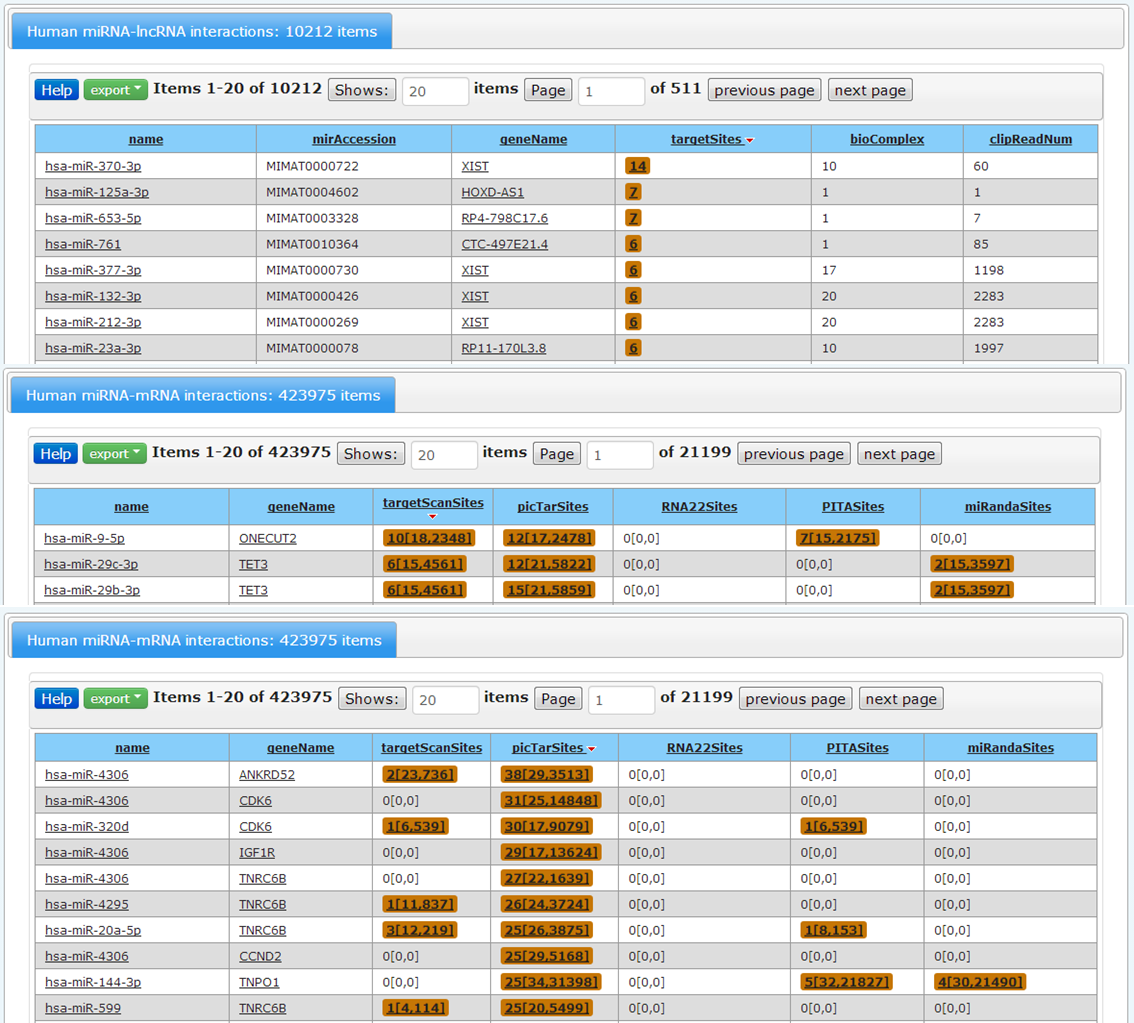


Figure S4A. Performing PTEN ceRNAs prediction in ceRNA regulatory networks from starbase v2.0


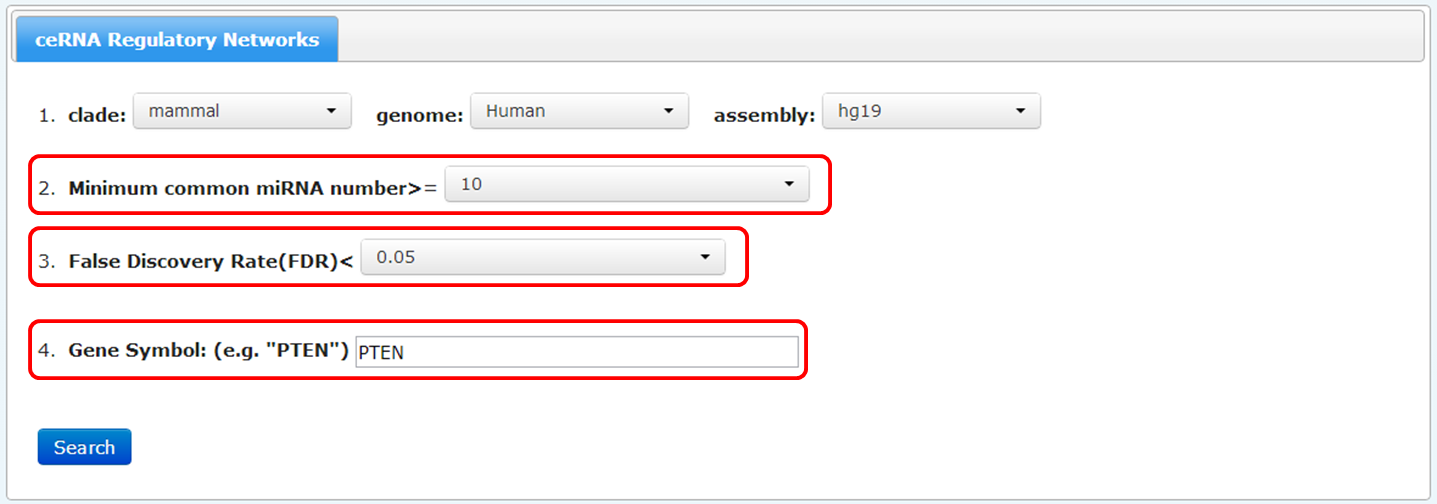


Figure S4B. The list of putative PTEN ceRNAs from the ceRNAs prediction method in starbase v2.0 platform


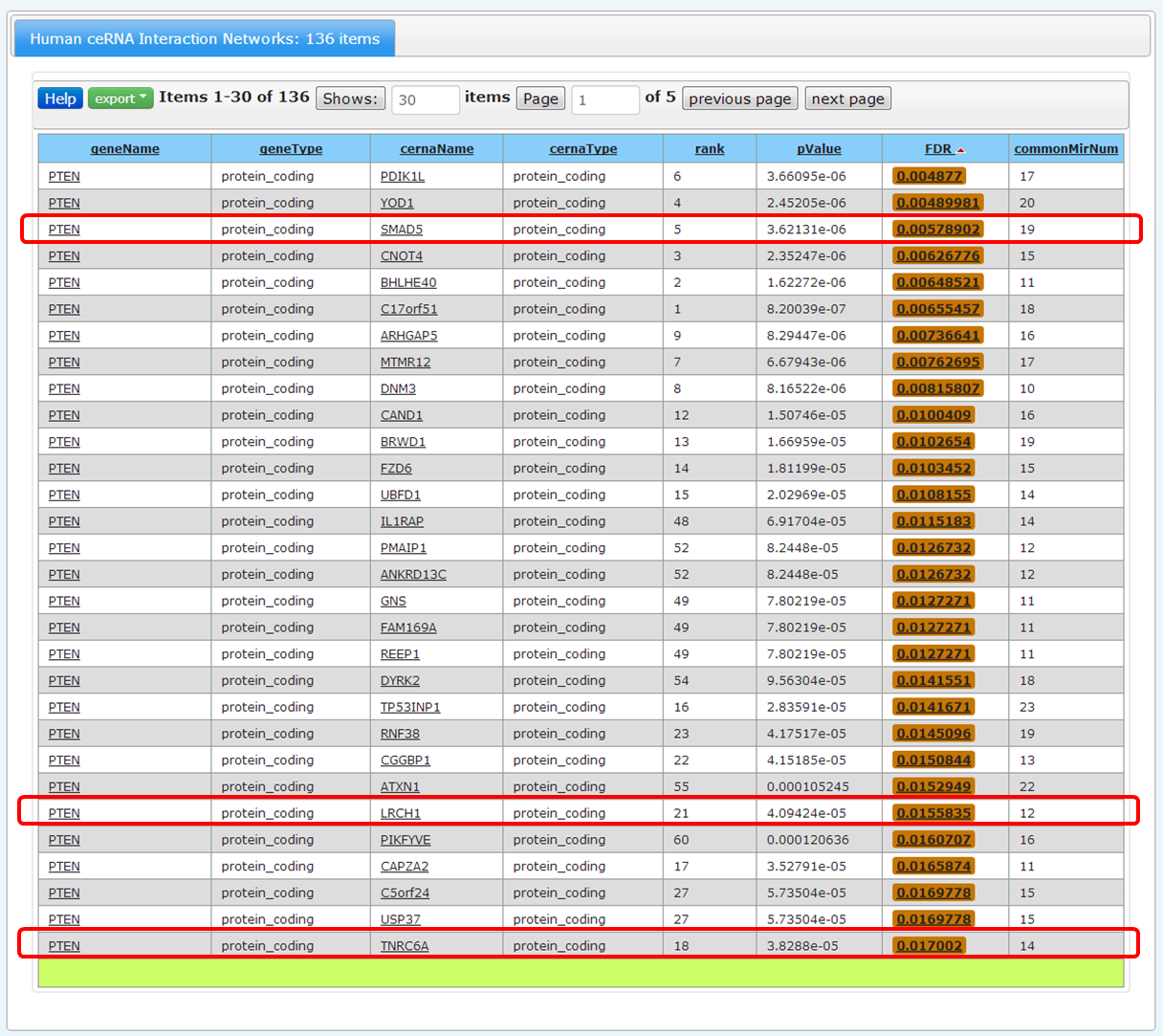


Figure S4C. The list of putative NFIB ceRNAs from the ceRNAs prediction method in starbase v2.0 platform


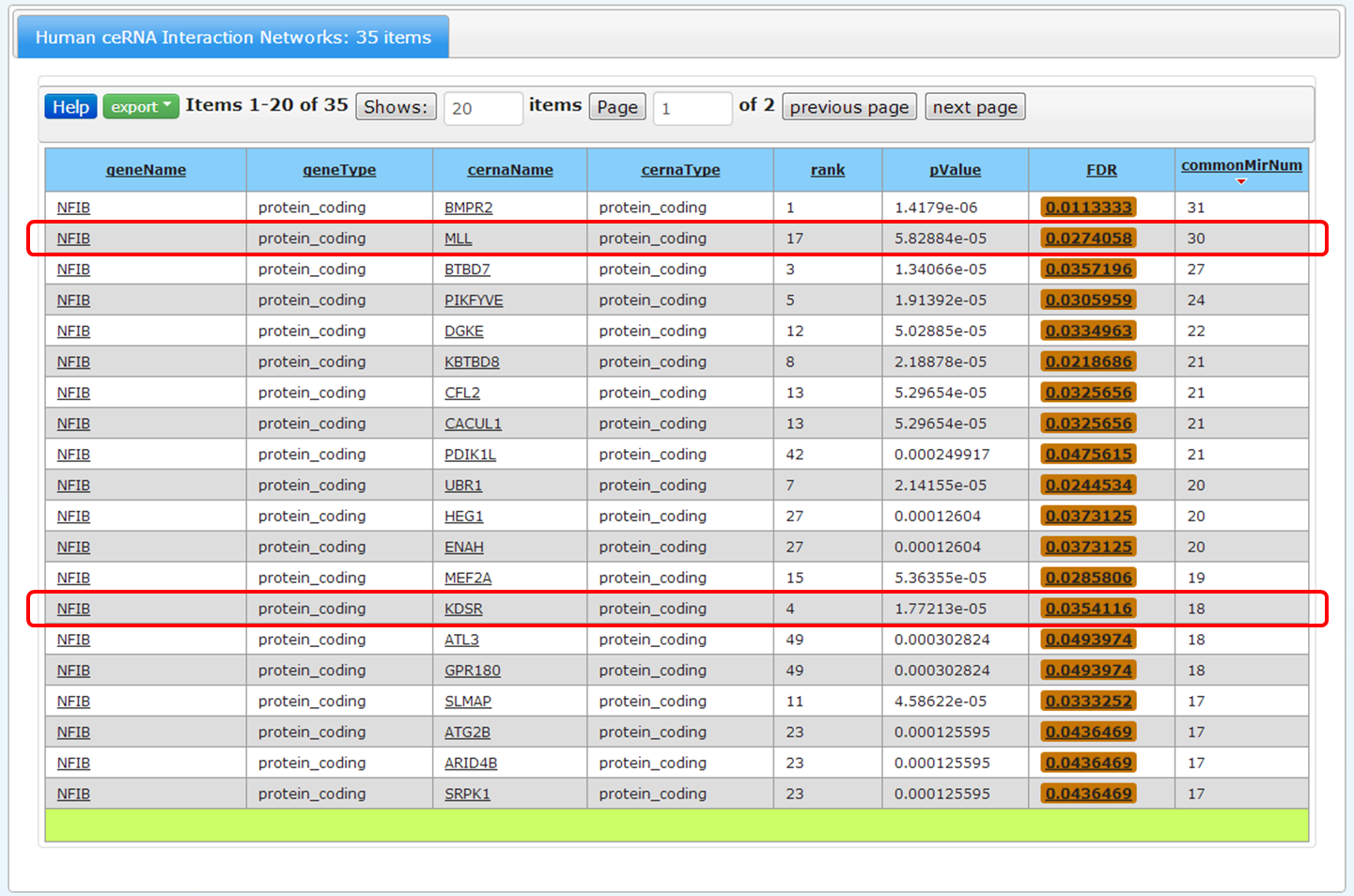


**References**

1. Lipchina, I., Elkabetz, Y., Hafner, M., Sheridan, R., Mihailovic, A., Tuschl, T., Sander, C., Studer, L. and Betel, D. (2011) Genome-wide identification of microRNA targets in human ES cells reveals a role for miR-302 in modulating BMP response. *Genes Dev*, **25**, 2173-2186.

2. Kishore, S., Jaskiewicz, L., Burger, L., Hausser, J., Khorshid, M. and Zavolan, M. (2011) A quantitative analysis of CLIP methods for identifying binding sites of RNA-binding proteins. *Nat Methods*, **8**, 559-564.

3. Memczak, S., Jens, M., Elefsinioti, A., Torti, F., Krueger, J., Rybak, A., Maier, L., Mackowiak, S.D., Gregersen, L.H., Munschauer, M. *et al.* (2013) Circular RNAs are a large class of animal RNAs with regulatory potency. *Nature*, **495**, 333-338.

4. Skalsky, R.L., Corcoran, D.L., Gottwein, E., Frank, C.L., Kang, D., Hafner, M., Nusbaum, J.D., Feederle, R., Delecluse, H.J., Luftig, M.A. *et al.* (2012) The viral and cellular microRNA targetome in lymphoblastoid cell lines. *PLoS Pathog*, **8**, e1002484.

5. Hafner, M., Landthaler, M., Burger, L., Khorshid, M., Hausser, J., Berninger, P., Rothballer, A., Ascano, M., Jr., Jungkamp, A.C., Munschauer, M. *et al.* (2010) Transcriptome-wide identification of RNA-binding protein and microRNA target sites by PAR-CLIP. *Cell*, **141**, 129-141.

6. Xue, Y., Ouyang, K., Huang, J., Zhou, Y., Ouyang, H., Li, H., Wang, G., Wu, Q., Wei, C., Bi, Y. *et al.* (2013) Direct conversion of fibroblasts to neurons by reprogramming PTB-regulated microRNA circuits. *Cell*, **152**, 82-96.

7. Karginov, F.V. and Hannon, G.J. (2013) Remodeling of Ago2-mRNA interactions upon cellular stress reflects miRNA complementarity and correlates with altered translation rates. *Genes Dev*, **27**, 1624-1632.

8. Haecker, I., Gay, L.A., Yang, Y., Hu, J., Morse, A.M., McIntyre, L.M. and Renne, R. (2012) Ago HITS-CLIP expands understanding of Kaposi's sarcoma-associated herpesvirus miRNA function in primary effusion lymphomas. *PLoS Pathog*, **8**, e1002884.

9. Riley, K.J., Rabinowitz, G.S., Yario, T.A., Luna, J.M., Darnell, R.B. and Steitz, J.A. (2012) EBV and human microRNAs co-target oncogenic and apoptotic viral and human genes during latency. *EMBO J*, **31**, 2207-2221.

10. Gottwein, E., Corcoran, D.L., Mukherjee, N., Skalsky, R.L., Hafner, M., Nusbaum, J.D., Shamulailatpam, P., Love, C.L., Dave, S.S., Tuschl, T. *et al.* (2011) Viral microRNA targetome of KSHV-infected primary effusion lymphoma cell lines. *Cell Host Microbe*, **10**, 515-526.

11. Helwak, A., Kudla, G., Dudnakova, T. and Tollervey, D. (2013) Mapping the human miRNA interactome by CLASH reveals frequent noncanonical binding. *Cell*, **153**, 654-665.

12. Zhang, C. and Darnell, R.B. (2011) Mapping in vivo protein-RNA interactions at single-nucleotide resolution from HITS-CLIP data. *Nat Biotechnol*, **29**, 607-614.

13. Leung, A.K., Young, A.G., Bhutkar, A., Zheng, G.X., Bosson, A.D., Nielsen, C.B. and Sharp, P.A. (2011) Genome-wide identification of Ago2 binding sites from mouse embryonic stem cells with and without mature microRNAs. *Nat Struct Mol Biol*, **18**, 237-244.

14. Loeb, G.B., Khan, A.A., Canner, D., Hiatt, J.B., Shendure, J., Darnell, R.B., Leslie, C.S. and Rudensky, A.Y. (2012) Transcriptome-wide miR-155 binding map reveals widespread noncanonical microRNA targeting. *Mol Cell*, **48**, 760-770.

15. Zisoulis, D.G., Lovci, M.T., Wilbert, M.L., Hutt, K.R., Liang, T.Y., Pasquinelli, A.E. and Yeo, G.W. (2010) Comprehensive discovery of endogenous Argonaute binding sites in Caenorhabditis elegans. *Nat Struct Mol Biol*, **17**, 173-179.

16. Sauliere, J., Murigneux, V., Wang, Z., Marquenet, E., Barbosa, I., Le Tonqueze, O., Audic, Y., Paillard, L., Roest Crollius, H. and Le Hir, H. (2012) CLIP-seq of eIF4AIII reveals transcriptome-wide mapping of the human exon junction complex. *Nat Struct Mol Biol*, **19**, 1124-1131.

17. Macias, S., Plass, M., Stajuda, A., Michlewski, G., Eyras, E. and Caceres, J.F. (2012) DGCR8 HITS-CLIP reveals novel functions for the Microprocessor. *Nat Struct Mol Biol*, **19**, 760-766.

18. Ascano, M., Jr., Mukherjee, N., Bandaru, P., Miller, J.B., Nusbaum, J.D., Corcoran, D.L., Langlois, C., Munschauer, M., Dewell, S., Hafner, M. *et al.* (2012) FMRP targets distinct mRNA sequence elements to regulate protein expression. *Nature*, **492**, 382-386.

19. Nakaya, T., Alexiou, P., Maragkakis, M., Chang, A. and Mourelatos, Z. (2013) FUS regulates genes coding for RNA-binding proteins in neurons by binding to their highly conserved introns. *RNA*, **19**, 498-509.

20. Hafner, M., Max, K.E., Bandaru, P., Morozov, P., Gerstberger, S., Brown, M., Molina, H. and Tuschl, T. (2013) Identification of mRNAs bound and regulated by human LIN28 proteins and molecular requirements for RNA recognition. *RNA*, **19**, 613-626.

21. Wilbert, M.L., Huelga, S.C., Kapeli, K., Stark, T.J., Liang, T.Y., Chen, S.X., Yan, B.Y., Nathanson, J.L., Hutt, K.R., Lovci, M.T. *et al.* (2012) LIN28 binds messenger RNAs at GGAGA motifs and regulates splicing factor abundance. *Mol Cell*, **48**, 195-206.

22. Sievers, C., Schlumpf, T., Sawarkar, R., Comoglio, F. and Paro, R. (2012) Mixture models and wavelet transforms reveal high confidence RNA-protein interaction sites in MOV10 PAR-CLIP data. *Nucleic Acids Res*, **40**, e160.

23. Baltz, A.G., Munschauer, M., Schwanhausser, B., Vasile, A., Murakawa, Y., Schueler, M., Youngs, N., Penfold-Brown, D., Drew, K., Milek, M. *et al.* (2012) The mRNA-bound proteome and its global occupancy profile on protein-coding transcripts. *Mol Cell*, **46**, 674-690.

24. Hoell, J.I., Larsson, E., Runge, S., Nusbaum, J.D., Duggimpudi, S., Farazi, T.A., Hafner, M., Borkhardt, A., Sander, C. and Tuschl, T. (2011) RNA targets of wild-type and mutant FET family proteins. *Nat Struct Mol Biol*, **18**, 1428-1431.

25. Sanford, J.R., Wang, X., Mort, M., Vanduyn, N., Cooper, D.N., Mooney, S.D., Edenberg, H.J. and Liu, Y. (2009) Splicing factor SFRS1 recognizes a functionally diverse landscape of RNA transcripts. *Genome Res*, **19**, 381-394.

26. Zarnack, K., Konig, J., Tajnik, M., Martincorena, I., Eustermann, S., Stevant, I., Reyes, A., Anders, S., Luscombe, N.M. and Ule, J. (2013) Direct competition between hnRNP C and U2AF65 protects the transcriptome from the exonization of Alu elements. *Cell*, **152**, 453-466.

27. Wang, Z., Kayikci, M., Briese, M., Zarnack, K., Luscombe, N.M., Rot, G., Zupan, B., Curk, T. and Ule, J. (2010) iCLIP predicts the dual splicing effects of TIA-RNA interactions. *PLoS Biol*, **8**, e1000530.

28. Konig, J., Zarnack, K., Rot, G., Curk, T., Kayikci, M., Zupan, B., Turner, D.J., Luscombe, N.M. and Ule, J. (2010) iCLIP reveals the function of hnRNP particles in splicing at individual nucleotide resolution. *Nat Struct Mol Biol*, **17**, 909-915.

29. Zund, D., Gruber, A.R., Zavolan, M. and Muhlemann, O. (2013) Translation-dependent displacement of UPF1 from coding sequences causes its enrichment in 3' UTRs. *Nat Struct Mol Biol*, **20**, 936-943.

30. Pandit, S., Zhou, Y., Shiue, L., Coutinho-Mansfield, G., Li, H., Qiu, J., Huang, J., Yeo, G.W., Ares, M., Jr. and Fu, X.D. (2013) Genome-wide analysis reveals SR protein cooperation and competition in regulated splicing. *Mol Cell*, **50**, 223-235.

31. Charizanis, K., Lee, K.Y., Batra, R., Goodwin, M., Zhang, C., Yuan, Y., Shiue, L., Cline, M., Scotti, M.M., Xia, G. *et al.* (2012) Muscleblind-like 2-mediated alternative splicing in the developing brain and dysregulation in myotonic dystrophy. *Neuron*, **75**, 437-450.

32. Ince-Dunn, G., Okano, H.J., Jensen, K.B., Park, W.Y., Zhong, R., Ule, J., Mele, A., Fak, J.J., Yang, C., Zhang, C. *et al.* (2012) Neuronal Elav-like (Hu) proteins regulate RNA splicing and abundance to control glutamate levels and neuronal excitability. *Neuron*, **75**, 1067-1080.

33. Ishigaki, S., Masuda, A., Fujioka, Y., Iguchi, Y., Katsuno, M., Shibata, A., Urano, F., Sobue, G. and Ohno, K. (2012) Position-dependent FUS-RNA interactions regulate alternative splicing events and transcriptions. *Scientific reports*, **2**, 529.

34. Licatalosi, D.D., Yano, M., Fak, J.J., Mele, A., Grabinski, S.E., Zhang, C. and Darnell, R.B. (2012) Ptbp2 represses adult-specific splicing to regulate the generation of neuronal precursors in the embryonic brain. *Genes Dev*, **26**, 1626-1642.

35. Wang, E.T., Cody, N.A., Jog, S., Biancolella, M., Wang, T.T., Treacy, D.J., Luo, S., Schroth, G.P., Housman, D.E., Reddy, S. *et al.* (2012) Transcriptome-wide regulation of pre-mRNA splicing and mRNA localization by muscleblind proteins. *Cell*, **150**, 710-724.

36. Liu, Y., Hu, W., Murakawa, Y., Yin, J., Wang, G., Landthaler, M. and Yan, J. (2013) Cold-induced RNA-binding proteins regulate circadian gene expression by controlling alternative polyadenylation. *Scientific reports*, **3**, 2054.

37. Jungkamp, A.C., Stoeckius, M., Mecenas, D., Grun, D., Mastrobuoni, G., Kempa, S. and Rajewsky, N. (2011) In vivo and transcriptome-wide identification of RNA binding protein target sites. *Mol Cell*, **44**, 828-840.
